# Current News and Opinion

**Published:** 1885-02

**Authors:** 


### Current News and Opinion.

### DINNER TO MR. TOMES.

Mr. Charles Tomes, of London, upon the occasion of his recent visit to America, was warmly welcomed at a dinner given in his honor at the Hotel Brunswick, New York, on Saturday, Dec. 27th, by the following friends ; Drs. Northrop, Dwinelle, Lord, Atkinson, Bronson, Kingsley, Jarvie, Hill, Myrick, Perry, Davenport, Bodecker, and Carr. Unfortunately, Drs. Francis and Cook were prevented from attending by illness. Under the combined influences of a table superbly decorated, and the solid enjoyment of the very perfection of a *menu*, each melted into his most genial mood, and contributed his quota of anecdote and joke, interspersed with lively reminiscences, professional and otherwise, for two or three hours, when Dr. Northrop, who presided, arose and gracefully proposed the first toast of the evening :—"The health of our honored guest." Mr. Tomes responded in a very happy manner, expressing the great pleasure that he experienced in again visiting America, and congratulated the members of the dental profession on both sides of the Atlantic for the hearty good-fellowship existing between them. Drs. Dwinelle and Kingsley then followed, recalling the many courtesies extended to them by Mr. John Tomes, the father of the guest, when they were in England twenty or more years ago. Nearly every one present forgot his diffidence and made a few remarks, one succeeding the other, until the limit of Saturday night was reached. Then, after cordially wishing Mr. Tomes a pleasant continuance of his visit among us, and a safe return home, guests and hosts separated. It was a peculiarly social occasion, and one that will not soon be forgotten.

C.

---

### MISSISSIPPI VALLEY ASSOCIATION OF DENTAL SURGEONS.

The forty-first annual meeting of the Mississippi Valley Association of Dental Surgeons will take place in the Lecture Room of the Ohio Dental College, at Cincinnati, at 10 o'clock, A. M., Wednesday, March 4th, 1885.

It is especially desirable that this meeting be unusually large, as it is expected that a number of papers on various subjects pertaining to the profession will be read by well-known writers, and in view of this the officers expect that every member will make it a point to assist by his personal presence. A most cordial invitation is extended to all members of the profession, no matter where they reside, to come and enjoy themselves, and profit by the interesting discussions that will take place.

PROGRAMME.

I.—Progress of Dental Education. II.—Recent Theories Concerning Caries of the Teeth. III.—New Remedies for the Treatment of Oral and Dental Diseases. IV.—Improvements in Prosthetic Dentistry. V.—New Methods and Materials for Filling Teeth. VI.—Incidents of Office Practice.

---

### AN ENGLISH ADVERTISEMENT.

TO DENTISTS.—The Managers of the North Surrey School District are prepared to appoint a duly qualified Dentist to attend to the teeth of the children at their School at Anerley. The salary offered will be £60 a year, and materials will be supplied. The time required for the performance of duties will be from about three to four hours a week. Information as to times of attendance, &c., can be obtained by calling personally upon the Superintendent at the Schools, Anerley. Forms, on which alone applications will be received, may be obtained from me, and must be returned not later than the 22nd instant.

88, High Street, Dorking, H. J. CHALDECOTT,
November 11th, 1884. Clerk to the Managers.

This reads strangely to an American. We are not accustomed to such an intelligent recognition by those in authority, of the necessity for early care of the teeth of children in public schools.

---

### GOLD MEDAL AWARDS TO UNITED STATES PRODUCTS AT INTERNATIONAL HEALTH EXHIBITION, LONDON, 1884.

Among the food products exhibited at the International Health Exhibition, London, 1884, from the United States, were *Beef Peptonoids* and *Maltine;* both of these preparations carried off the only Gold Medal and highest Award against numerous competitors in their respective classes. All food preparations were critically analyzed at this Exhibition by a jury composed of the best chemists in the country.—*London Lancet.*

### HOW TO KEEP CIDER SWEET.

When the saccharine matters by fermentation are being converted to alcohol, if a bent tube be inserted air-tight into the bung, with the other end in a pail of water, to allow the carbonic acid gas evolved to pass off without admitting any air into the barrel, a beverage will be obtained that is fit nectar for the gods.

A handy way is to fill a cask nearly up to the wooden faucet, when the cask is rolled, so that the bung is down. Get a common rubber tube and slip it over the end of the plug in the faucet, with the other end in the pail. Then turn the plug so the cider can have communication with the pail. After the water ceases to bubble, bottle or store away.—*Farm, Field and Fireside.*

---

### CHLOROFORM *vs.* ETHER.

Dr. T. Gaillard Thomas stated at the late meeting of the American Gynecological Society, that he had substituted chloroform for ether in all cases in which there is any kidney disease. Ether has a peculiarly irritant action upon renal structures in a pathological state. In chronic Bright's disease, ether frequently induces uræmia, by suppression of the urinary secretion.

---

### TO AVOID NAUSEA.

Making a patient keep his eyes closed while recovering from ether is a great aid in preventing sickness; for, owing to the patient feeling giddy, any object at which he looks appears to sway from side to side, and this by itself is sometimes enough to produce a feeling akin to sea-sickness, even in those who have not been anæsthetized.—*Med. and Surg. Reporter.*

---

### A SPECIMEN.

All professional illiteracy is not among the dentists, as the following death certificate, that recently accompanied a body from the West to Buffalo, will amply prove:

I hearby certyfy that the deseased come to her death by Dialeipyra or Feaver & the desease is not contachious.

P—— D——, M. D.,
Attending Physition.

### TENNESSEE DENTAL ASSOCIATION.

The Nineteenth Annual Meeting of the Tennessee Dental Association will be held in Nashville, beginning Tuesday, the 24th day of February, 1885, at 10 A. M., in the Lecture Hall of the University of Tennessee.

---

### A WELCOME HOME.

Dr. Northrup gave a dinner at the Hotel Brunswick, on Saturday evening, Jan. 17th, to a few friends, to welcome Dr. Bogue, who had just returned from Paris. C.

---

### THE ADMINISTRATION OF NITROUS OXIDE.

Pregnant women, even as far advanced as eight months, take nitrous oxide well. I may here mention the case of two patients, who were nursing, and in whom the shock from the extraction of a tooth, without the gas, stopped the milk secretion. Subsequently, having to undergo a similar operation under like circumstances, they determined to take the gas, and in neither instance did any derangement of the function of lactation occur. Children with chorea, and people who have had hemiplegia, take the gas well. In phthisical patients, if the mischief is extensive, some caution is necessary, as in these cases the anæsthesia deepens after the removal of the face-piece, so that it is neither necessary nor advisable to start with any deep amount of insensibility. It is perfectly safe to administer this agent to epileptic patients. I have met with several cases where the extraction of a tooth without the gas has produced an epileptic attack, but I have never seen one occur when gas has been given. Great age is no bar to the administration of nitrous oxide, my oldest patient, a lady, having reached the age of ninety-four.—*Dr. Woodhouse Braine in Brit. Med. Jour.*

---

### PRESCRIPTIONS USEFUL FOR NERVOUSNESS.

Tincture of valerian, tincture of hyoscyamus, equal parts. Mix. Dose, a teaspoonful in water, three times a day.

Valerinate of zinc, one drachm; ext. of conium, one drachm; ext. gentian, two drachms. Mix and divide into sixty pills. Dose, one pill three or four times a day.

Tincture valerian, tincture hyoscyamus, spirits lavender, each one ounce; paregoric, two drachms. Mix. Dose, teaspoonful every three hours till rest is procured.

Tincture cinchona, tinct. valerian, tinct. hyoscyamus, spirits lavender, each one ounce; spirits camphor, one-half ounce. Mix. Dose, teaspoonful three times a day.

---

### OUR RULES.

Being determined, if possible, that no one shall be able to say that the Independent Practitioner has been sent to him against his wish, we print our "Rules of Publication" in this issue. We would not, under any circumstances, send a copy to any one unless it was our understanding that it was desired; and in order that none escape seeing our rules we insert them here, with the request that if there be one among our many readers who is not willing to pay the trivial sum of $2.50 per year, he let us know.

TERMS—The subscription price is Two Dollars and Fifty Cents per year in *advance.*

REMITTANCES should be made to Dr. C. E. Francis, Treas., No. 33 West 47th Street, New York, by post-office money order or registered letter.

ADDRESS—Always give the name of the county as well as that of the post-office. Removals should be promptly reported, that the address may be changed and loss to the subscriber prevented. In doing so give the former address also.

DISCONTINUANCES—*No discontinuances will be made until arrearages are paid, and instructions given.* This is a postal law sustained in all courts to prevent serious loss to publishers. Refusal to remove a journal from the post-office does not exempt the subscriber from liability for its cost, so long as arrearages are unpaid. When arrearages are paid and a subscriber wishes to have his journal discontinued, he should so notify the publishers by card or letter.

FRAUD—Removing from any neighborhood and failing to notify a publisher to stop sending a periodical to that address, is regarded by postal laws as *prima facie* evidence of fraud, unless the accumulated arrearages are paid on demand. Without the protection of such a law publishers are exposed to very heavy loss.

The journal is mailed on the first day of each month. If you fail to receive it within a reasonable length of time thereafter, please notify the Buffalo office by postal card.

(41.) How can I utilize waste amalgam, old fillings, etc.? Also, where can I find instructions for making amalgams, calculated to meet the various conditions of teeth requiring that material. STUDENT.

(42.) In trying to remove a tooth pulp I was unfortunate enough to break a Swiss broach, the end of which remains fast in the root. Will some one please let me know how to get it out? R.

(43.) Sometimes parties come to me for artificial teeth, who have, perhaps, one or two teeth, more or less firm, remaining in the mouth, which they are not willing to part with. I generally advise them to have such teeth extracted, but they sometimes prefer to keep them. Am I not right in so advising them, and is it not better in the end to make a "clean sweep" in such cases? LABORATORY.

(44.) How many States have laws for restricting the practice of dentistry? Are the laws in the different States similar in requirements? By replying will much oblige. S. B.

(45.) Of what utility is the sulphate of potassa that many use for mixing plaster of paris in taking impressions? Has it any other effect than to make the plaster set quicker? I wish that some of our chemists would answer this. P. B. R.

(46.) "Hydrochlorate of Cocaine." Will some one please inform me concerning the physiological action of this preparation on animal tissue? INQUIRER.

### Answers.

R. H. B. (37.) If the teeth referred to have "never given the slightest annoyance whatever," better by all means "leave them unmolested." If they have stood the test for "fifteen years," they may never give trouble. To open them would incur a risk, and if unpleasant results followed, your patient would blame you, and you would blame yourself for your pains. F.

BOSTON. (38.) Your statement is not sufficient to enable one to judge understandingly. It is possible that the neuralgic pain proceeds from the teeth, but not probable, unless the pain can be determined as having its inception since the teeth became diseased. Let me ask a few questions that will suggest their own connection with the case. Does treatment of the teeth produce any change in the neuralgic condition? Are the pains of a true neuralgic character—that is, distinctly paroxysmal and intermittent, and induced by fatigue, malaise, and nervous exhaustion? Do they follow the course of any particular nerve? Are there tender spots anywhere upon the face, notably at the supra or infra-orbital, or mental foramens? Is there any particular soreness along the course of any nerve after a violent paroxysm or attack of the malady? Is there any pain from violent thermal changes inside the mouth? You have already stated that the pains are unilateral, and this may or may not be a valuable diagnostic sign. M. D.

CINCINNATUS. (39.) Perhaps the tooth which is "neither sore nor painful," but is "becoming loose," has no antagonizing fellow; or a scale of calculus may have formed on the root beneath the gum. Your description is not very clear. Leave the temporary stopping as it is for a time, and see what comes of it, but be sure that the gum is kept healthy and free from extraneous collections. To diagnose all cases with absolute correctness is hardly to be expected. Who can do it? "GOTHAMITE."

PENNSYLVANIAN. (40.) Sure enough! Why *will* so many dentists deal in misnomers? It is about time such errors were corrected. It is as stupid to call a tooth pulp a "nerve", as to call a first molar a "six-year-old molar." IOWA.

